# A web-based control system for traditional street lighting that uses high-pressure sodium lamps

**DOI:** 10.1016/j.heliyon.2021.e08329

**Published:** 2021-11-05

**Authors:** Anurak Thungtong, Chanchai Chaichan, Korakot Suwannarat

**Affiliations:** a School of Engineering and Technology, Walailak University, Thasala, Nakhon Si Thammarat, 80160, Thailand; b Center for Digital Technology, Walailak University, Thasala, Nakhon Si Thammarat, 80160, Thailand

**Keywords:** Street lighting control, High pressure sodium lamp, Internet of things, NB-IoT, Microcontroller, Web application

## Abstract

Street lighting is a critical component of any city's infrastructure. On the other hand, the street lighting system consumes a significant amount of electricity. As a result, many technologies and studies are being developed to reduce the energy cost of street lighting. While the majority of the proposed ideas for reducing the energy cost of the street lighting system are based on light emitting diode lamps, they are not suitable for high-pressure sodium lamps, which continue to dominate in developing countries. Moreover, the high initial cost, difficulty of installation and maintenance, reliability, and service lifetime are all significant barriers to the practical implementation of these ideas. This paper presents a web-based control system for traditional street lighting systems that still employs high-pressure sodium lamps. The proposed idea converts existing modules of the conventional controller, which are photo switches, into IoT devices. The web application on the server then manages and controls the devices. The web application allows users to create a schedule for turning off the lights during the late-night hours to save energy. The system's advantages include its low cost, ease of installation, and maintenance. The proposed system is useful for roads or areas with low traffic density at late night. This system has been validated at Walailak University, Thailand.

## 1 Introduction

The street lighting system is an important infrastructure in cities around the world. It improves citizen safety on the roads and increases security in public places. A street lighting system boosts economic growth by extending the amount of time people spend outside at night. Unfortunately, one of the major contributors to significant energy consumption is the street lighting system. The production of electrical energy produces more carbon dioxide emissions, accelerating the phenomenon of the greenhouse effect. As a result, improving the efficiency of street lighting is an important responsibility for cities all over the world.

In the literature, several methods for improving the energy efficiency of street lighting systems have been proposed. The first method is to replace the traditional high pressure sodium (HPS) lamp with a light emitting diode (LED) lamp. The LED lamp uses significantly less energy than the HPS lamp. Furthermore, as technology advances, the cost of LED lamps falls dramatically. As a result, because the payback period occurs before the end of the LED lifetime, replacing HPS with LED lamps is economically feasible [1]. Moreover, a study compared the environmental performance of LED and HPS lamps and discovered that the life cycle of HPS lamps has a 9 percent higher environmental impact than LED lamps. While the environmental impact of HPS and LED manufacturing is 13 percent and 4 percent, respectively. As a result, it is estimated that by 2020, LED technology will have a 45 percent lower environmental impact than HPS technology [2]. According to some studies, LED lamps are the most promising energy-saving light source because cities can save up to 40–60 percent on energy costs by replacing conventional light bulbs with LED lamps [3, 4].

When combined with other technologies, LED lamps enable even greater efficiency. The first set of concepts uses wireless sensor networks composed of sensors, microcontrollers, and wireless communication modules to turn on and off LED lights in response to road occupancy detected via light dependent resistors (LDR), infrared sensors (IR), or ultrasonic sensors. On/off lighting control is intended for groups of lampposts linked by a wireless network such as ZigBee. The sensor is installed at the first lamppost in this scheme. When the sensor detects approaching vehicles or pedestrians, it transmits a signal to the microcontroller, instructing it to turn on the forward group of lamps and turn off the trailing lampposts as the vehicles pass [5, 6, 7, 8, 9]. Later, the sensor network was connected to the web-based controller via internet of things (IoT) technology, enabling remote monitoring and management of the lighting system. Numerous wireless communication technologies have been proposed for IoT-based street lighting systems, including ZigBee [10], WiMAX [11], GSM [12, 13, 14], LoRa [15, 16], and NB-IoT [17]. Cloud-based platforms for street lighting control have also been proposed [18, 19, 20]. The IoT-based street lighting system has been enhanced to facilitate the construction of a smart city. Numerous works have combined the street lighting system with other components for this purpose, including a faulty streetlight detector [21], emergency buttons installed on light poles that send an emergency signal to the police station [22], camera units that manage the traffic signal [23], special sensors for traffic counting [24], human detectors that can alert authorities to probable theft at odd hours [25], and smart poles that equip cameras to detect and report any motion events [26]. The dimming performance of LED lamps is another important technology that researchers are interested in. Basic dimming systems adjust LED lamp brightness to one of many preset settings based on traffic density and environmental variables sensed by LDR, infrared, rain, or fog sensors [27, 28, 29]. To further improve energy efficiency, complex algorithms ranging from statistical models [30, 31, 32, 33] to artificial neural network models [34, 35] have been utilized to manage light intensity based on traffic volume. Additionally, photovoltaic or solar cell technology is an emerging technology that plays an important role in street lighting systems. Solar cells are used as an alternative energy source in smart standalone street lighting systems, which are made up of LED light lamps, road occupancy sensors, microcontrollers, and smart algorithms that control solar charging as well as LED brightness [36], and a communication module for connecting the system to the internet [37, 38]. Smart standalone street lighting systems offer significant energy savings [39, 40] and are now commercially available. Finally, sophisticated algorithms and models were employed to create regulations and plans for increasing the energy efficiency of the street lighting system [41, 42, 43, 44].

Although many ideas for reducing the energy consumption of street lighting have been proposed, there are some challenges and limitations to consider. While current LED technologies outperform HPS lamps in almost every way, and the majority of energy-saving ideas are geared toward LED lamps, HPS lamps continue to be dominant in street lighting applications in developing countries. In Thailand, for example, the Department of Highways' standards for street lighting systems have not been updated to include LED technologies. Hence, most existing HPS lamps are not being replaced with LEDs, and even new installations of street lighting systems continue to rely on HPS. As a result, most energy-saving ideas developed for LED lamps are not directly applicable to HPS lamps. Controlling the lamps based on traffic density, for example, is an intriguing technique for LED lamps. The HPS lamp, on the other hand, requires a few minutes of warm-up time before it can operate at full light intensity, and it also requires some time for the reigniting process after it has been turned off. Therefore, the HPS lamp does not respond to sensor signals instantly.

Other issues with existing techniques for practical implementation include high initial costs, installation and maintenance difficulties, reliability, and service lifetime [45]. Due to these factors, many ideas for reducing the energy consumption of street lighting systems proposed in the literature are still in the laboratory setup [16, 18, 46, 47]. Even commercial products may be unpopular if the initial cost is prohibitively high. Dimming ballasts, for example, are designed to reduce the light intensity of HPS lamps [48, 49]. There are two ways to dim HPS lamps. First, electronic ballasts can be used to implement continuous dimming. Hotel lobbies, shopping malls, and sports arenas are examples of continuous dimming applications. Step dimming is another dimming method that is commonly used with magnetic ballasts. HPS lamps can be dimmed in two to three steps using this method. Step dimming ballasts, when used in conjunction with a timer, can dim the lamp power by 50 percent in the late-night hours. Unfortunately, the material and installation costs of a dimming ballast and timer are expensive. As a result, dimming ballasts are rarely used in standard street lighting applications.

**Figure 1:**
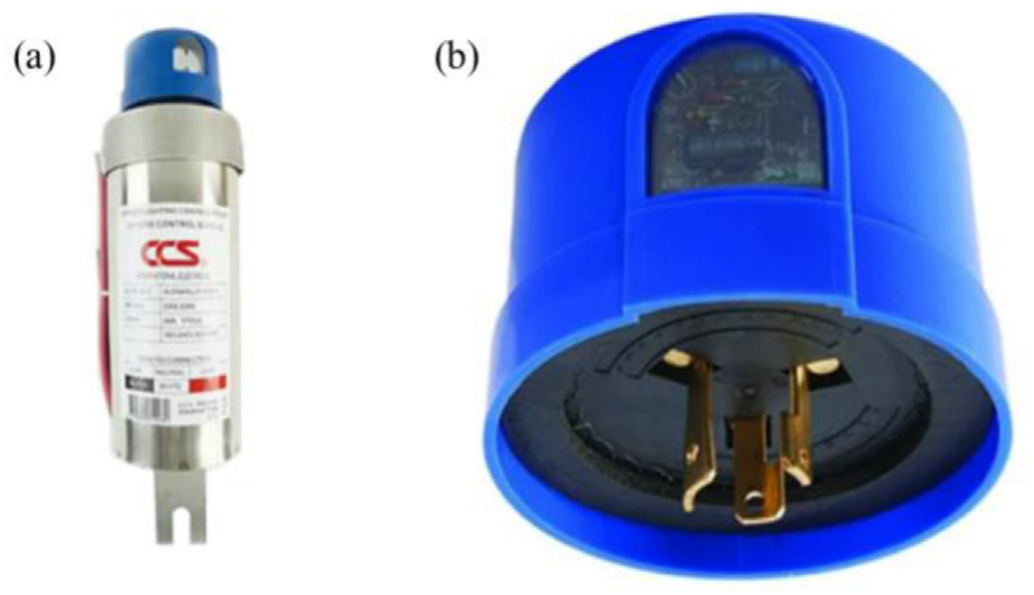
In the existing street lighting system, there is an LDR photo switch and a 220 V, 60–100 A relay presented in subfigure (a). The photo switch module can be disconnected from the relay unit as illustrated in subfigure (b).

With all these challenges, appropriate energy-saving techniques that are compatible with existing HPS lamps are essential. The existing street lighting system with HPS lamps uses a standard street lighting control unit to turn on or off the lamps. The control unit is made up of two modules: a photo switch (LDR sensor) and a 220 V, 60–100 A relay, both of which are separable, as shown in Figure 1. In general, the photo switch module turns on the lamp when the light intensity is less than 20 Lux and turns it off when the light intensity is greater than 40 Lux. As a result, the light will be on for approximately 12 h per day. This paper proposes a method for saving more energy by using a web-based controller to turn off HPS lamps during the late-night hours. To reduce costs while increasing ease of installation and maintenance, we propose modifying an existing photo switch module to be an IoT switch and controlling the switch state of the device via a web server. To accomplish this, we replaced the LDR sensor in the photo switch module with a reed switch and used a microcontroller to control its switch state. The NB-IoT module connects the microcontroller to a central server. Instead of light sensors, this architecture switches the HPS light lamps on and off based on sunset and sunrise information received from a web application programming interface (API). The pre-programmed timetable is also used to turn off the lamps at night. The user-friendly GUI is intended to intelligently manage a large number of end devices. The sections that follow describe the method and the results of its implementation in Thailand.

## 2 Methods

### 2.1 System architecture

**Figure 2:**
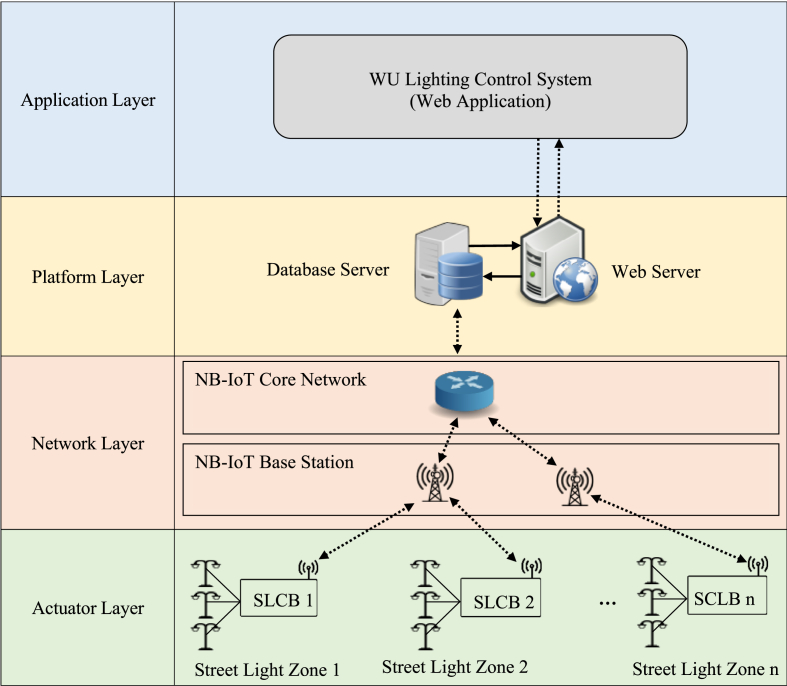
The proposed street lighting control system is composed of four major components: the actuator layer, the network layer, the platform layer, and the application layer.

**Figure 3:**
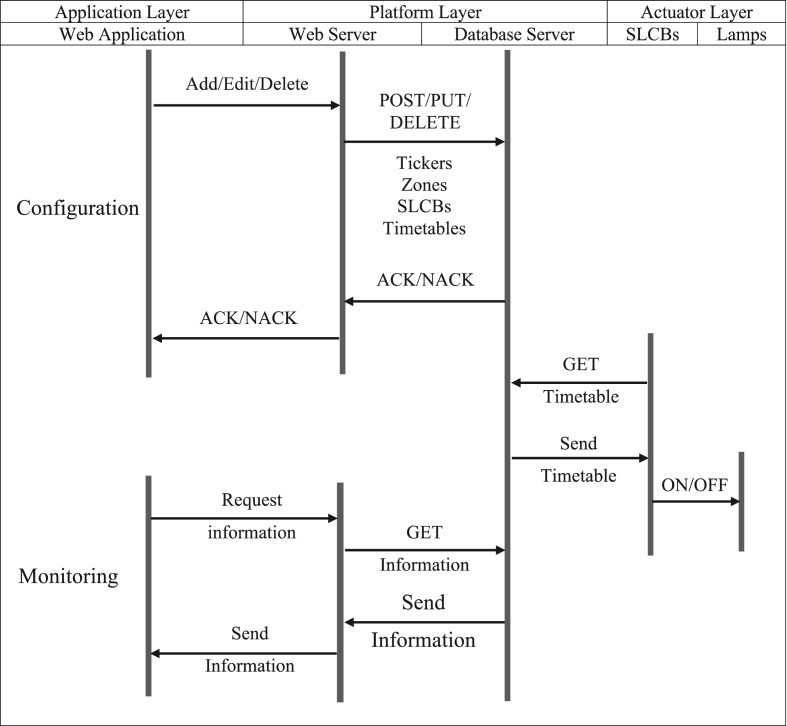
System flow diagram shows communication protocol between layers.

The proposed control system for street lighting with HPS lamps employs a client-server architecture comprised of four major components, as illustrated in Figure 2. The communication protocol between each component was designed using the system flow diagram depicted in Figure 3. The actuator layer contains a microcontroller module for controlling the lamps, and a wireless communication module for connecting the microcontroller to the internet. The network layer is the communication system infrastructure available in the area. The platform layer contains a database and a webserver that maintains all configuration and status of the system. Finally, the application layer enables users to configure and monitor the system (add/remove/group devices, set on/off timetable, and monitor system status). The following sections present more details about each component.

#### 2.1.1 Actuator layer

A conventional street lighting system operates by switching on and off a group of ten to twenty lampposts in response to the amount of light detected by an LDR sensor in the photo switch module. The photo switch module sends the on/off signal to the relay which is connected to the electrical controller unit of the traditional street lighting system. As illustrated in Figure 1, the photo switch module is detachable from the relay. This design is advantageous for us because it enables us to convert a traditional control unit into an IoT device by modifying only the photo switch module and leaving the rest of the system unchanged. In this project, the transformation simply starts by replacing the LDR sensor in the photo switch module with a reed switch, an electromechanical switching device that opens and closes contacts using simple magnet interaction. To turn the power supply on and off, the reed switch requires digital logic signals 0 and 1, which can be provided by a microcontroller. Due to the remote location of the controller unit in the traditional street lighting system, a wireless communication module is required to transmit an on/off status from a web server to the microcontroller. As a result, the actuator layer consists of three modules: a modified switch, a microcontroller, and a wireless communication module. A collection of these modules is referred to as a street lighting control box (SLCB).

Although there are numerous microcontrollers available, we chose the ESP32 microcontroller for this project because it is a low-cost, low-power microcontroller optimized for remote devices. The ESP32 microcontroller has several peripheral input/output interfaces, including digital general i/o pins, ADCs (analog-to-digital converters), DACs (digital-to-analog converters), I^2^C (Inter-Integrated Circuit), UART (universal asynchronous receiver/transmitter), CAN 2.0 (Controller Area Network), SPI (Serial Peripheral Interface), I^2^S (Integrated Inter-IC Sound), RMII (Reduced Media-Independent Interface), PWM (pulse width modulation), and more. However, this project uses only the digital pin for controlling the reed switch and a UART for communicating with the wireless communication module. Additionally, the ESP32 supports Wi-Fi (802.11n @ 2.4 GHz with a maximum data rate of 150 Mbit/s) and Bluetooth v4.2 BR/EDR, making setup and configuration easier.

Wireless communication technologies such as Wi-Fi, Bluetooth, LoRaWAN, and NB-IoT are frequently used in IoT projects. In general, when selecting wireless technologies for IoT systems, it is necessary to consider the power consumption, coverage range, and data rate of the communication technologies. However, because the SLCBs are installed on lampposts that are always connected to the power supply, power consumption is unaffected. Table 1 compares the coverage range and data rate of the four wireless communication standards mentioned previously. According to the table, Wi-Fi and Bluetooth technologies have a high data rate but a short communication range [50]. However, in this project, the SLCBs are installed along the street up to 2.5 km from the server. As a result, Wi-Fi and Bluetooth are ineffective, but LoRaWAN and NB-IoT are candidates because their coverage ranges exceed 10 km [51].

**Table 1:** Coverage range and data rate of wireless communication technologies.

Wireless Technology	Coverage Range	Data Rate
Wi-Fi	95 m	54 Mbps
Bluetooth 4.x	5–30 m	54 Mbps
LoRaWAN	>15 km.	Up to 5.47 kbps
NB-IoT	>10 km.	20 kbps for uplink, 200 kbps for downlink

**Figure 4:**
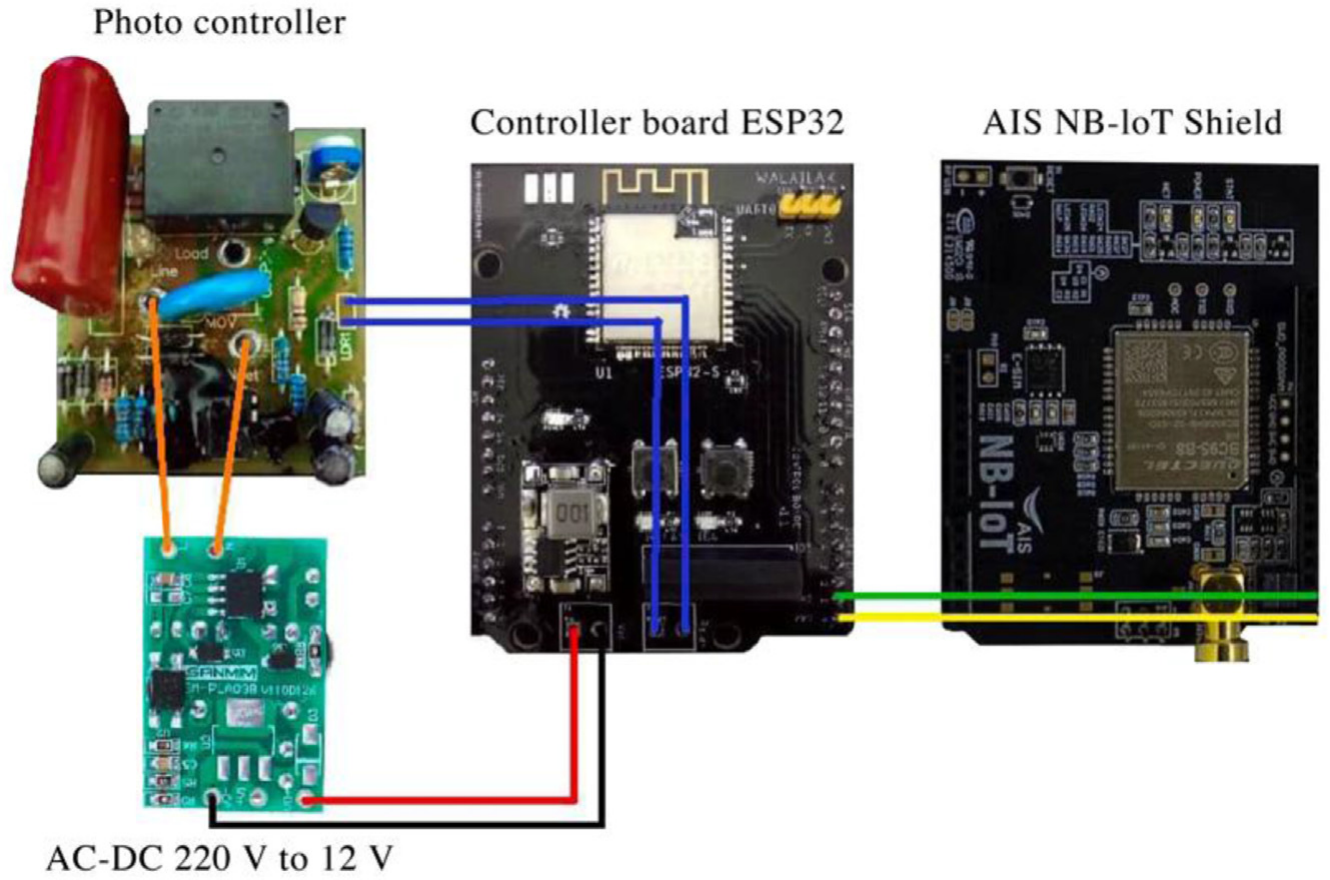
Connection diagram of the photo controller module, power supply module, ESP32 microcontroller module, and AIS NB-IoT shield in an SLCB unit.

**Figure 5:**
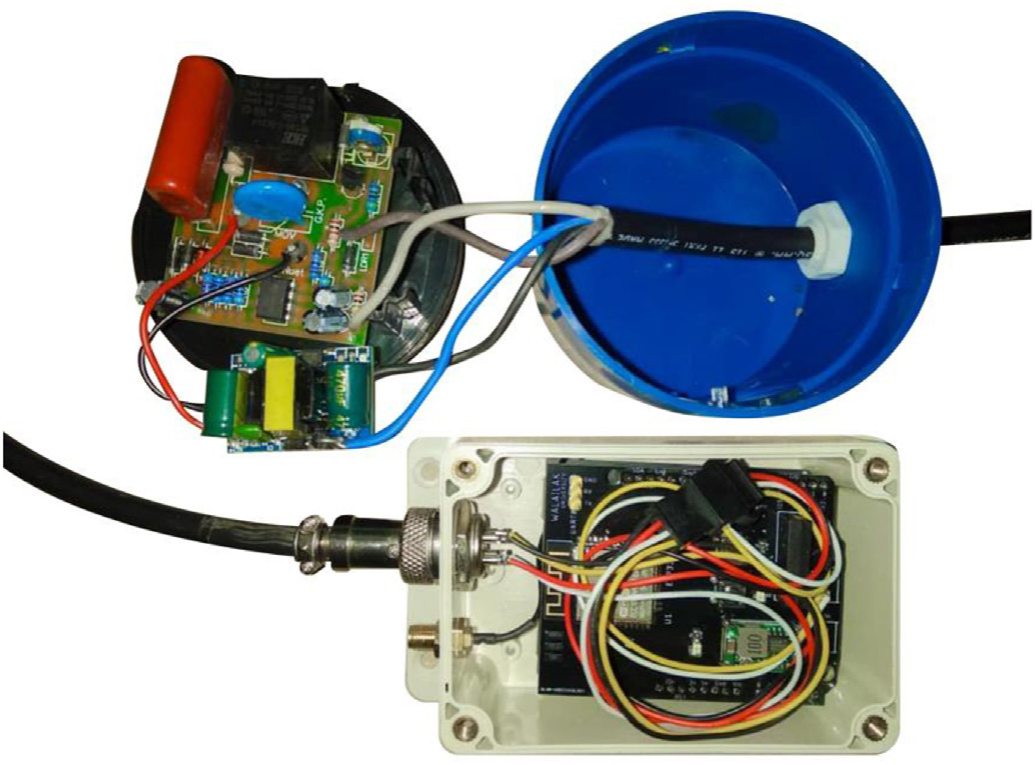
An SLCB unit is constructed of two boxes connected by four-core cables. The round box includes the modified photo switch module, which will be connected to the relay unit. The square box contains the microcontroller and NB-IoT modules. This box will be placed in the electrical control case of the traditional street lighting system. An antenna will be mounted externally to receive the full signal.

The proposed system will be validated in Thailand as part of this project. As a result, the choice of a wireless communication module is based on signal coverage in the chosen area. The National Broadcasting and Telecommunications Commission in Thailand manages the LoRaWAN network, which uses the frequency range of 920–925 kHz for LoRaWAN communication [52]. LoRaWAN is a wireless network architecture with a star topology that uses a centralized gateway to communicate between the server and end nodes [53]. Unfortunately, the gateways are not yet installed in the city where we intend to test the system. NB-IoT is a Low Power Wide Area Network (LPWAN) technology standard that is designed for transmitting and receiving small amounts of data from remote locations by utilizing a subset of the 3G/4G/LTE standard. NB-IoT technology is currently provided in Thailand by Advance Info Service (AIS) and TRUE telephone carriers [54]. However, the AIS network covers 98 percent of Thailand. As a result, AIS NB-IoT was chosen as the communication system for this project. The UART communication interface connects the NB-IoT to the ESP32 microcontroller. The integration of the modified photo switch, ESP32 microcontroller, and NB-IoT module is shown in Figure 4. Figure 5 depicts the finished product of an SLCB unit.

#### 2.1.2 Network layer

The NB-IoT module communicates with the internet via the NB-IoT base station and core network of the AIS internet service provider. In general, the NB-IoT module's IP address is a dynamic IP, whereas the server's IP address is fixed. As a result, the microcontroller module is programmed to communicate with the server via the GET command. Every five minutes, the microcontroller sends three identical request packages to the server using User Datagram Protocol (UDP) in order to report its switch status and request for the on/off timetable. The microcontroller then controls the reed switch based on the timetable received. If the communication is not successful, the microcontroller will operate the system using the most recent timetable. This enables the SLCBs to function normally even if the server connection is lost.

#### 2.1.3 Platform layer

We stored all the data in the MySQL database management system. The database server accepts connections from all SLCBs, which will communicate with it continuously to update their switch statuses and request their timetable. If the database server does not receive a connection from an SLCB within five minutes, the connection status of this SLCB is set to offline. Additionally, the database server is connected to the web server, which is configured on the Ubuntu operating system with the Apache HTTP server. The web server queries data from the database server to display on the web application and sends all configurations, such as the on/off timetable of all SLCBs, to the database server for storage. We use Python and Javascript to manage access to the database via the RESTful API [55]. This technique is advantageous when multiple applications share a single database.

#### 2.1.4 Application layer

We created a web application for configuring and monitoring the system using PHP and JavaScript. The website's address is http://lcs.wu.ac.th/ (log in is required for security reasons). The web application facilitates users to configure and monitor the street lighting system.

**Figure 6:**
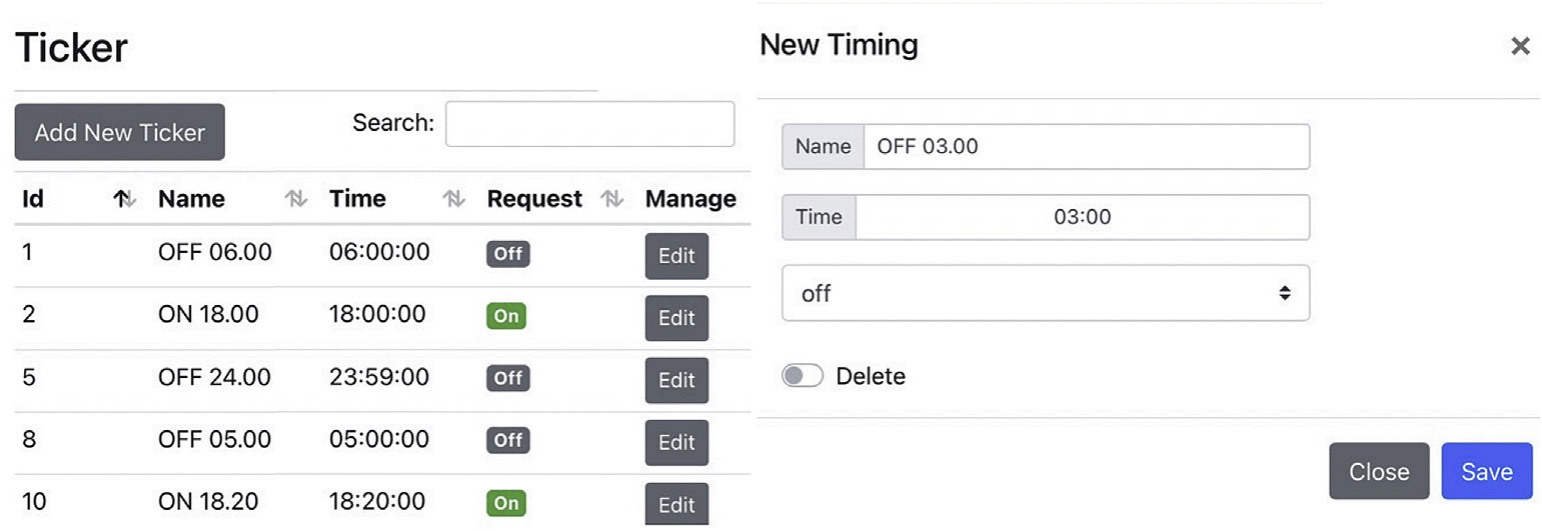
The Ticker dialog is designed for creating time and request (on/off). These tickers are used later to specify the on or off time of zones or devices.

**Figure 7:**
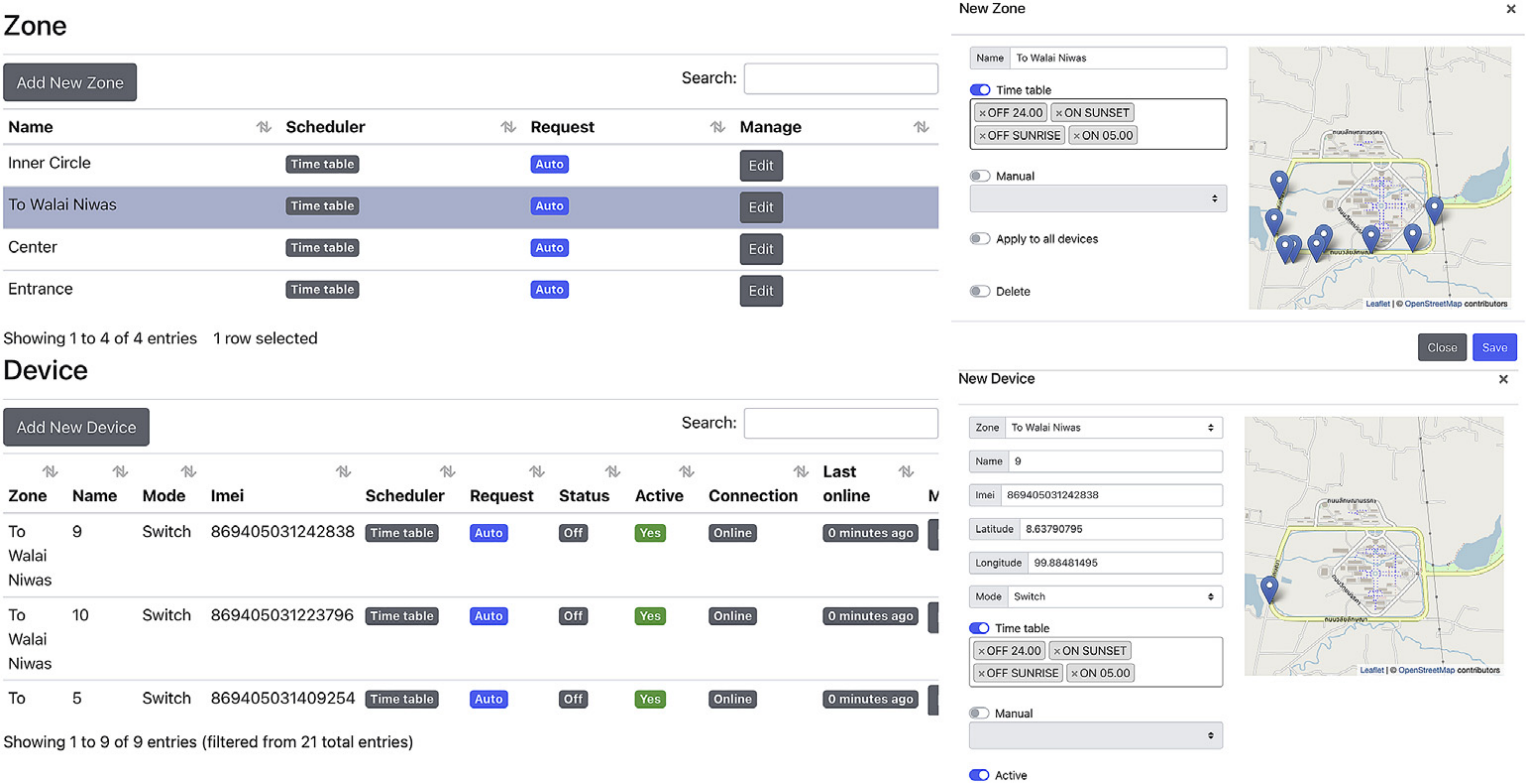
The manage window is divided into two sub-dialogs. The Zone dialog is used to create zones and schedule timetables for all devices (SLCBs) in the zone. The Device dialog is used to add devices to the currently selected zone. This dialog specifies the NB-IoT device's name, IMEI number, and location. The device's timetable is also specified independently here.

In the configuration process, users create tickers which are timestamps and the desired switch states in the Ticker dialog shown in Figure 6. These tickers will be used to generate the on/off timetable in the future. Following that, users must create a zone which is a collection of SLCBs located in the same area and intended to be operated identically. This step can be accomplished by utilizing the Zone dialog as depicted in Figure 7. The users are then prompted to add additional devices (SLCBs) to the selected zone by specifying the device's name, imei number, and GPS coordinates. Finally, the users must generate the on/off schedule for all devices using either the zone dialog in which all SLCBs in that zone will be assigned the same timetable, or the device dialog in which all devices can be assigned a timetable independently.

**Figure 8:**
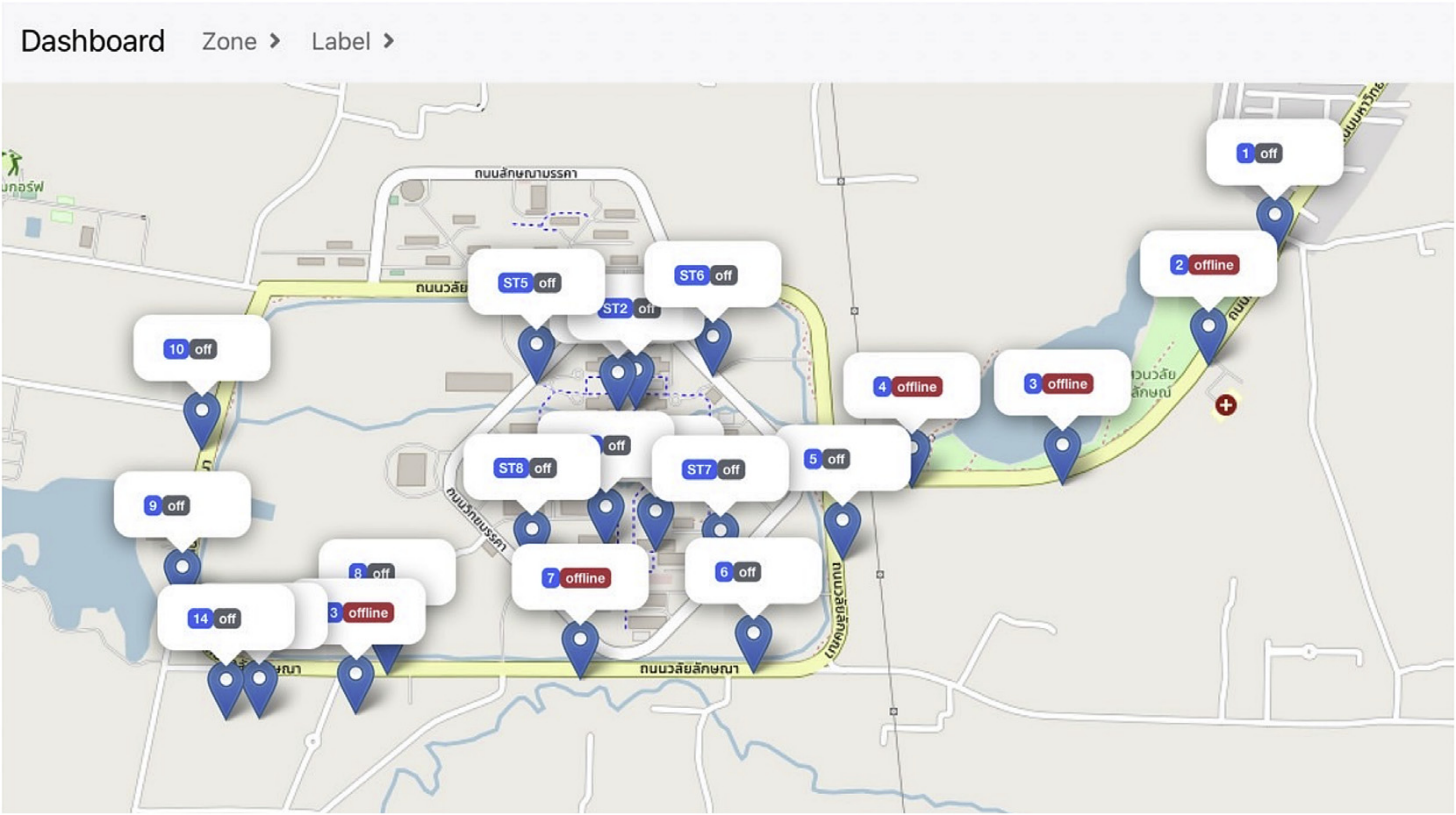
Dashboard window presents name, status, and location of the SLCBs installed at Walailak University.

**Figure 9:**
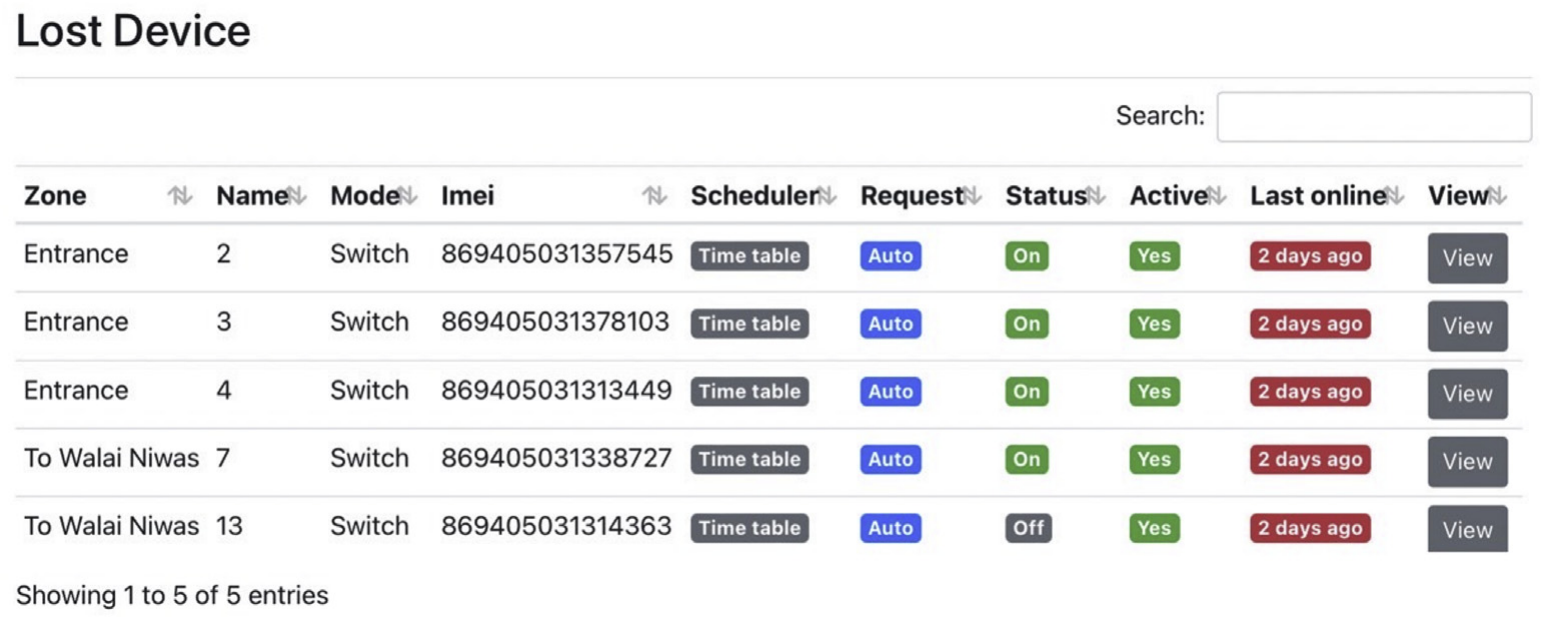
The Lost Device table shows all offline SLCBs and the last time that they are online.

**Figure 10:**
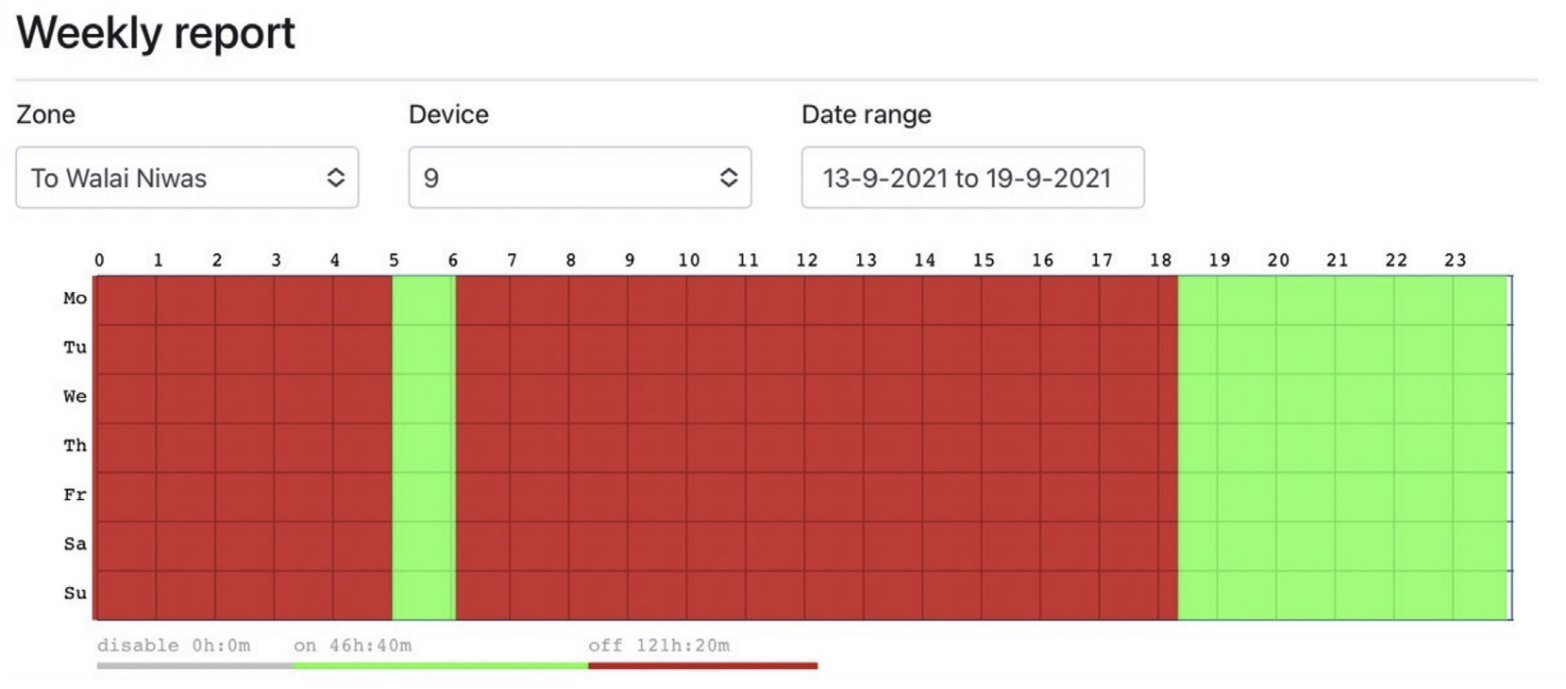
SLCBs status presented in weekly report is designed for user to investigate the system performance.

In the monitoring process, the web application offers users to view system status in three windows. The dashboard, depicted in Figure 8, displays the name, status (on/off/offline), and location of all SLCBs. The Lost Device window, shown in Figure 9, collects information about all SLCBs that are currently offline, as well as the last time the devices were online. Finally, the recorded status of SLCBs is displayed in the Weekly report window (Figure 10). These windows assist technicians in diagnosing problems and properly maintaining the system.

### 2.2 Installation

We implemented the proposed system at Walailak University, Thailand's largest university in terms of land area. The university is run as a residential institution, with approximately 5,000 students living on campus. The university operates a street lighting system via 53 control units. Each unit is equipped with ten to twenty HPS lamps. As a result, the campus is illuminated by approximately 2,500 lamps. Previously, the control units functioned as switches for turning on and off the light lamps in response to the sun's intensity. The average annual energy cost is approximately 133,000 US dollars, or approximately five percent of the university's total energy cost.

We converted twenty-one conventional photo switch modules into smart IoT switches. These switches control approximately 400 lamps distributed throughout the university in four zones: the Center, Inner Circle, Entrance, and Walai-niwas. Each zone contains four, four, four, and nine SLCBs respectively. Figure 8 depicts the location of the SLCBs. Prior to onsite installation, we configured the devices using the ESP32 microcontroller's wireless communication module. Then, the name, GPS coordinator, zone, and timetable of the devices were set up on the website. Following that, the onsite installation was as simple as three steps: removing the conventional photo switch module, replacing it with the modified switch module, and placing the microcontroller box in the control unit with the antenna outside. These steps take only 15 min for one SLCB.

### 2.3 Timetable design

All the lamps were programmed to turn on at sunset and off at sunrise. The sunset and sunrise timestamps are obtained from the website sunrise-sunset.org/api by sending the API code to the website along with the latitude and longitude. It is important to note that the time received is Coordinated Universal Time (UTC), which is seven hours behind Thailand.

We determined the optimal time to turn off the lamps at night by counting the number of vehicles passing through each zone. The observation lasted one week. The average number of vehicles per hour in each zone is shown in Table 2. We turn off the lamps if the number of vehicles is less than ten. As a result, after turning on the lamps at sunset, we set the timer to turn them off from 11 PM to 5 AM for the center zone, 12 AM–5 AM for the inner circle zone, 2–4 AM for the entrance zone, and 12 AM–4 AM for the Walai-Niwas zone. Following these times, the lamps will be turned on until sunrise.

**Table 2:** The average number of vehicles per hour (rounded to integer) passing through four zones.

Time	Center	Inner Circle	Entrance	Walai-Niwas
18.00–19.00	176	185	241	207
19.00–20.00	101	128	215	180
20.00–21.00	92	134	158	136
21.00–22.00	64	99	124	102
22.00–23.00	28	63	99	55
23.00–24.00	9	34	82	19
24.00–01.00	7	8	33	6
01.00–02.00	5	7	15	4
02.00–03.00	3	1	8	2
03.00–04.00	4	2	6	3
04.00–05.00	5	6	13	12
05.00–06.00	11	19	30	19

## 3 Results

We evaluated the performance of SLCBs in terms of hardware stability and communication quality between NB-IoT and the server by measuring the percent offline time of all devices. During the first three days after installation, the status of SLCBs (on, off, or offline) was recorded every five minutes. The percentage of offline time was calculated, and the results are shown in Table 3. Based on the table, SLCBs can be classified into two groups. Fourteen SLCBs (66.67 percent of all SLCBs) are in the first group, with zero percent offline time (SLCBs ST1-ST8, 3, 4, 7, 8, 10, 13, 14). Although the second group is not constantly online, the percentage of time spent offline is quite low, with an average of 3.28 percent (SLCBs 1, 2, 5, 6, and 9). This characteristic has no effect on system performance. These findings demonstrate the reliability of the AIS NB-IoT shield and ESP32 microcontroller modules.

**Table 3:** Percent offline time of SLCBs in the first three days.

SLCB Names	Day 1	Day 2	Day 3	Average
ST 1	0	0	0	0
ST 2	0	0	0	0
ST 3	0	0	0	0
ST 4	0	0	0	0
ST 5	0	0	0	0
ST 6	0	0	0	0
ST 7	0	0	0	0
ST 8	0	0	0	0
1	3.6	1.2	1.2	2
2	1.2	3.0	0.60	1.6
3	0	0	0	0
4	0	0	0	0
5	4.8	7.8	1.8	4.8
6	1.8	0.6	3.0	1.8
7	0	0	0	0
8	0	0	0	0
9	1.2	0	17.4	6.2
10	0	0	0	0
13	0	0	0	0
14	0	0	0	0
23	0	0	0	0

We tracked energy-saving results a month after installation using data from the power meters installed at all control units. Table 4 compares the monthly power consumption of street lighting when it was controlled by IoT switches versus standard photo switches. According to this data, we can save up to 36.3 percent of energy by turning off the lamps at night. The total amount of money we can save per month is 1327.8 USD. Because the total cost of the project is approximately 3,000 USD (cost of electronic components, NB-IoT modules, annual data service charge, and a personal computer used as a server), the payback period for this project is 3000 (USD)/ 1327.8 (USD/month) = 2.26 months.

**Table 4:** Monthly power usage of 21 SLCBs before and after modifying photo switches to IoT-based switches.

SLCB Names	Photo switch (KWH)	IoT Switch (KWH)	Energy Saves (KWH)	Energy Saves (percent)	Money Saves (USD)
ST 1	1021	498	523	51.2	68.2
ST 2	1840	918	922	50.1	120.3
ST 3	464	229	235	50.6	30.6
ST 4	337	169	168	49.9	21.9
ST 5	2036	1203	833	40.9	108.6
ST 6	4585	2714	1871	40.8	244.0
ST 7	2939	1731	1208	41.1	157.6
ST 8	2908	1695	1213	41.7	158.2
1	573	485	88	15.4	11.5
2	1457	1218	239	16.4	31.2
3	1784	1488	296	16.6	38.6
4	645	535	110	17.1	14.4
5	639	428	211	33.0	27.5
6	566	385	181	32.0	23.6
7	232	155	77	33.3	10.1
8	1177	791	386	32.8	50.4
9	953	633	320	33.6	41.8
10	329	218	111	33.7	14.5
13	1325	884	441	33.3	57.6
14	1128	743	385	34.1	50.2
23	1098	738	360	32.8	47.0
Total	28036	17858	10178	36.3	1327.8

## 4 Discussion and conclusion

The goal of this research is to propose a feasible control method that will save energy for the conventional street lighting system. The cost and difficulty of installation and maintenance are the primary factors we consider when designing the control system. The key feature of our proposed system is that we convert the photo switch modules into IoT devices that are controlled by a web-based controller. Despite its simplicity, this method is appropriate the existing street lighting system, which employs HPS lamps. Our system has a very low initial cost and a very short payback period. Additionally, installation and maintenance are extremely simple. The installation process for each SLCB takes about 15 min, which includes replacing the traditional photo switch with the modified one. If a modified switch fails, technicians can replace it with a standard photo switch, and the lighting system is immediately restored. The modified switch can then be repaired and reinstalled when it is completed. Furthermore, using our user-friendly interface, users can divide SLCBs into zones and create a timetable for each zone or even each SLCB independently. When the timetable needs to be changed, the web application makes it simple. Additionally, the GUI sends an alert when any SLCBs become offline and maintains a history of all SLCB statuses. These features are extremely beneficial to the maintenance team.

Our proposed controller saves energy for the street lighting system by allowing users to turn off the lamps to shorten their operating time. As a result, it is not appropriate for areas that require continuous lighting throughout the night, but it is useful if the area allows for some hours of darkness. For instance, a street with low traffic density at late-night, such as a rural road or a private area. However, to increase safety, our proposed system can be used in conjunction with standalone street lighting lamps, which can be installed on lampposts near intersections or areas that require illumination overnight.

In conclusion, we propose a method for controlling a traditional street lighting system in which the lamps are turned on and off using a standard photo switch module. The proposed method converts the photo switch module into an IoT switch controlled by the central server. The web application facilitates users to turn off the lights during late-night hours to save energy. The proposed system is inexpensive, easy to install and maintain, and dependable. It is appropriate for rural roads or any areas where traffic density is very low during the late-night hours. The proposed system was validated at Walailak University, Thailand. By controlling the lamps with the proposed web-based controller rather than photo sensors, the proposed system saves up to 36.3 percent of energy costs.

## Declarations

### Author contribution statement

Anurak Thungtong: Conceived and designed the experiments; Performed the experiments; Analyzed and interpreted the data; Wrote the paper.

Chanchai Chaichan: Performed the experiments; Contributed reagents, materials, analysis tools or data.

Korakot Suwannarat: Conceived and designed the experiments; Performed the experiments; Analyzed and interpreted the data; Wrote the paper.

### Funding statement

Anurak Thungtong was supported by 10.13039/501100010034Walailak University (WU62229).

### Data availability statement

Data included in article/supplementary material/referenced in article.

### Declaration of interests statement

The authors declare no conflict of interest.

### Additional information

No additional information is available for this paper.
